# Endophytic fungal species *Nigrospora oryzae* and *Alternaria alternata* exhibit antimicrobial activity against gram-positive and gram-negative multi-drug resistant clinical bacterial isolates

**DOI:** 10.1186/s12906-023-04157-8

**Published:** 2023-09-15

**Authors:** Asiphe Fanele, Sizwe I. Ndlovu

**Affiliations:** 1 Discipline of Medical Microbiology, School of Laboratory Medicine and Medical Sciences, College of Health Sciences, University of KwaZulu-Natal, Durban, South Africa; 2 Department of Biotechnology and Food Technology, Doornfontein Campus, University of Johannesburg, Johannesburg, South Africa

**Keywords:** Endophytic fungi, *Sclerocarya birrea*, Multidrug-resistant pathogens, Antibacterial activity, 2-fluorobenzoic acid heptadecyl ester

## Abstract

**Background:**

The emergence of multidrug-resistant pathogens and the lack of new antimicrobial drugs is a major public health concern that needs urgent and innovative solutions. Endophytic fungi living in unique niches such as in endosymbiosis with plants are increasingly drawing attention as alternative sources of novel and chemically diverse compounds with unique mechanisms of action.

**Methods:**

In the present study, ten endophytic fungi isolated from the medicinal plant, *Sclerocarya birrea* were screened for bioactivity against a panel of indicator bacteria. Three bioactive endophytic fungi (strains P02PL2, P02MS1, and P02MS2A) were selected and identified through ITS-rDNA sequencing. The whole broth extracts of the three selected isolates were further screened against contemporary drug-resistant bacterial pathogens. This was followed by partial purification by solid phase extraction and GC–MS analysis of bioactive fractions.

**Results:**

The bioactive endophytic fungi were identified as *Alternaria alternata* species (strains P02PL2 and P02MS1) and *Nigrospora oryzae* (strain P02MS2A). The whole broth extracts from *N. oryzae* P02MS2A exhibited a MIC of one μg/mL and 16 μg/mL against gram-negative, MDR *Pseudomonas* 5625574 and gram-positive MRSA 25775 clinical isolates, respectively. After partial purification and GC–MS analysis of whole broth extract from *A. alternaria* PO2MS1, 2-fluorobenzoic acid heptadecyl was putatively identified as the active compound in fraction C of this extract. This compound was also putatively identified in fraction E of *A*. *alternata* P02PL2, fraction B of *A. alternata* P02MS1 and fraction B of *N. oryzae* P02MS2A, and interestingly, all these fractions retained activity against the two MDR clinical isolates.

**Conclusion:**

The putative identification of 2-fluorobenzoic acid heptadecyl compound showing a broad-spectrum of activity, more especially against gram-negative MDR contemporary pathogens is highly encouraging in the initiative at developing novel drugs to combat multi-drug resistance.

**Supplementary Information:**

The online version contains supplementary material available at 10.1186/s12906-023-04157-8.

## Introduction

The increasing problem of antimicrobial resistance (AMR) coupled with the emergence of new pathogens has been identified by the World Health Organisation (WHO) as one of the top ten global priorities that need urgent attention [1]. Over the recent decades, there has been a continued paucity of new and effective antimicrobial drugs which continues to be a threat to the success of modern medicine including major surgeries such as transplants, and cancer chemotherapy as the risk of infection is increased during these procedures [2, 3]. It is estimated that at least 700 000 people die each year from infections caused by drug-resistant pathogens [4]. If there are no sound interventions for the AMR problem, it is projected that the number could rise to 10 million people by 2050 [5, 6]. The WHO has set four innovation criteria to be fulfilled by new compounds. These include (1) the absence of known cross-resistance to existing antibiotics, (2) a new class of antibiotics, (3) a new target or binding site, and (4) new modes of action [7]. However, compounds that do not meet all these criteria may still be clinically valuable in specific treatment conditions [8].

To ensure a steady flow of new antimicrobial drug candidates into the clinical pipeline, there are increasing research efforts on hit identification and hit-to-lead optimization programs [9]. To do this, the early stages of drug discovery and development must be improved since they are critical for identifying and validating innovative candidate drugs capable of combating antimicrobial resistance [10]. Atanasov et al. [11] highlighted natural-product-based hit compounds as a strategy to find new bioactive molecules. This strategy is supported by the fact that natural products have been the most important source of new drugs during the last 40 years; around 60% of all known novel chemical entities in the field of antibacterial are microbial natural products or semi/synthetic derivatives of natural products [12]. Microbial natural products cover a wider area of chemical space with highly diverse structures compared to synthetic molecules. In addition, they exhibit interesting drug-like features which includes lower cLogP values indicating a higher hydrophobicity and higher structural rigidity which makes them highly likely to target new binding sites such as protein–protein interactions [11]. The reactivated research efforts to discover new drugs from microbial natural products are mainly driven by biodiversity mining, i.e., isolation of diverse fungal and bacterial from unique environmental niches using innovative isolation and cultivation techniques to increase the chances of identifying new chemical structures. This is typically followed by bioactivity screening that use standardized tests to assess the bioactivity of fungal or bacteria crude extracts (compounds) against a panel of test microorganisms and chemical characterization using chromatographic tools such as Gas Chromatography-Mass Spectrometry (GC–MS) for volatile compounds or Liquid Chromatography-Mass Spectrometry (HPLC–MS) for non-volatile compounds [13–15].

Endophytic fungi are microorganisms that colonize and proliferate in the inner plant tissues without causing any apparent harm [16]. Instead, the endophytic fungi promote the plant's growth and aid the plant to withstand biotic and abiotic factors, including stress tolerance and drought resistance by synthesizing a plethora of secondary metabolites [17, 18]. In addition, these secondary metabolites also give the plant a competitive advantage against competing pathogens in the natural environment [19, 20]. The diverse nature of environmental conditions that the plant must endure suggests that the secondary metabolites produced as a response represent interesting biological properties that could be explored in medicine, agriculture, and industry. Hence, the fungal kingdom is well known as a source of interesting properties such as antibacterial compounds (cephalosporin C) [21], antifungal compounds (enfumafungin and amphotericin B) [22, 23], immunosuppressant (statins) [24], etc. While the fungal kingdom has been largely explored for active compounds [25], the potential of fungi found in unique niches such as endophytic fungi has been less explored but holds great potential to yield diverse structures that can meet the urgent need for novel and highly potent compounds to target the emergency of novel microbial pathogens and emerging drug resistance profiles [26].

More recently, an increasing interest in exploring endophytic fungi has been sparked by the discovery that they can produce the same or similar compounds to their plant hosts. In this way, the ethnobotanical knowledge of medicinal plants provides a promising lead to isolate fungi with interesting properties [27]. This also means that endophytic fungi could serve as a sustainable alternative to plant-produced secondary metabolites and will also enable large-scale production of the secondary metabolites using the already well-established fermentation systems [28]. Subsequently, the isolation of fungal endophytes for the synthesis of secondary metabolites associated provides an alternative way to search for bioactive organic compounds (secondary metabolites) with minor disruption of plants [29]. The bioactive compounds extracted from plants-fungal endophytes have gained attractiveness in the natural products and pharmaceutical research field worldwide as an alternative source of therapeutic agents that can assist in combating the increasing antimicrobial resistance problem [30]. In this study, we assessed the bioactivities of crude extracts from three endophytic fungal isolates against selected microorganisms such as *Staphylococcus aureus* ATCC 25923, *S. aureus* CS (clinical strain), methicillin resistant *S. aureus* (MRSA) 25775, and multidrug resistant (MDR) *Pseudomonas* 5625574).

## Material and methods

### Isolation of endophytic fungi

Ten endophytic fungi (P02ML1, P02ML2, P02ML3, P02PL1, P02PL2, P2PS1, P02MS1, P02MS2A, P02MS2B, and P02MS3) were isolated from the leaves and bark of *Sclerocarya birrea*. This medicinal plant was collected from the South Coast region of the Durban Municipality, KwaZulu-Natal (30º 03’ S; 30º 53’ E). The plant was sent to the University of KwaZulu-Natal (UKZN) School of Life Science Herbarium for taxonomic identification and registration (voucher number 18234). Isolation of endophytic fungi was done following a modified protocol described by Shara et al. [31]. Briefly, the leaves and stem of the medicinal plant were washed twice in distilled water. The surfaces were then sterilized in 70% (v/v) ethanol for one minute, then in sodium hypochlorite (3% v/v available chlorine) for four minutes before immersing them again in 70% (v/v) ethanol for another minute. The final wash was done three times in distilled water and air-dried. Small cut pieces (5–7 mm) of the leaves and bark were then aseptically transferred onto potato dextrose agar (PDA, Oxoid, UK) and malt extract agar (MEA, Oxoid, UK) plates supplemented with 100 μg/mL ampicillin (Merck, South Africa) to prevent bacterial growth. To confirm the absence of epiphytic fungi, the final water wash was also plated on PDA and MEA agar plates supplemented with ampicillin. The plates were cultured in the dark at 25ºC for five days. This was followed by subsequent subculturing until pure single fungal cultures were retained and stored in 50% glycerol at -80ºC for further use [32].

### Macroscopic and microscopic identification of fungal endophytes

The fungal isolates were revived from the glycerol stocks by culturing on either malt extract agar (MEA, Oxoid, UK) or potato dextrose agar (PDA, Oxoid, UK) supplemented with 100 μg/mL ampicillin for seven days at 25ºC in the dark. The morphological identification of endophytic fungi isolates was first performed through analysis of macroscopic and microscopic characteristics. Briefly, a segment of each pure fungal endophyte hyphal tip was cut off from the culture using a sterile blade. Each segment was cut into the size of a 6 mm^2^ disk and re-inoculated into a fresh antibiotic-free malt extract agar (MEA) growth medium. All isolates were incubated at 25ºC for seven days. Phenotypic characterization of each fungal isolate was done following the method described by Salvamani and Nawawi [33], specifically paying attention to mycelia growth diameter, growth rate, texture, the color of the colony, and the shape of the growing colony of each fungal strain [33].

The microscopic characterization of endophytic fungi isolates was done based on the slide culture technique method described by Rodriguez-Tudela and Aviles [34]. Briefly, a wet mount of each fungal endophyte isolate was prepared using a lactophenol cotton blue stain. This was done by placing a drop of lactophenol cotton blue stain into the microscope slide, then a growing mycelium of selected endophytic fungi isolate was scraped from fresh growing culture into a microscopic slide using a sterile needle to form a suspension of mycelia and lactophenol cotton blue stain. The suspension was covered with a coverslip and observed under a light microscope [34].

### Molecular identification of endophytic fungi

About 100 mg of mycelia from the fresh fungal cultures were collected for genomic DNA extraction. Genomic DNA was isolated using the Norgen Plant/Fungi DNA isolation kit (25240, Norgen Biotek, Thorold, ON, Canada) as per the manufacturer’s instructions. The purity and concentration of the extracted DNA were assessed with a NanoDrop 2000c Spectrophotometer (Thermo Fisher Scientific, South Africa). Amplification of the internal transcribed spacer (ITS) region was done by polymerase chain reaction (PCR), where the forward primer, ITS1 (5′-TCCGTAGGTGAACCTGCGG-3′), and the reverse primer, ITS4 (5′-TCCTCCGCTTATTGATATGC-3′) were used to amplify the ITS sequence region from the isolated fungal DNA. The PCR mix constituted of Phusion hot start II High-Fidelity 1X Master Mix (Thermo Fischer, Carlsbad, CA, USA), ITS1 primer (0.5 µM), ITS4 primer (0.5 µM), and 50 ng fungal DNA.

The PCR was conducted under the following conditions: 98ºC for 30 s, 30 cycles of 98ºC for 10 s, 45ºC for 30 s, 72ºC for 1 min, and 72ºC for 2 min. The quality of the extracted DNA samples was assessed using horizontal agarose gel electrophoresis analysis on 1% (w/v) agarose gel (SeaKem® LE Agarose). The bands formed on the agarose gel were visualized using Uvitec Platinum computer-based gel imaging system (Uvitec®). The PCR products were then purified using the PureLink® PCR Purification kit, Invitrogen (Thermo Fisher Scientific, Carlsbad, CA, USA) as per the manufacturer’s instructions. After purification, the amplicons for each fungal isolate were quantified using a NanoDrop 2000c (Thermo Scientific, South Africa) and sent to Inqaba Biotech (Pretoria, South Africa) for internal transcribed spacer (ITS) sequencing.

The quality assessment of the ITS sequences was performed using Snap Gene Viewer version 6.1.2 followed by the generation of a consensus sequence for each isolate. ITS sequences were compared using the Basic Local Alignment Tool (BLAST) application of the nucleotide database of the National Centre for Biotechnology Information (NCBI) with fungal ITS genes on the database. The isolates P02MS1 and P02PL2 were respectively identified as *Alternata alternaria* (accession numbers OP629950 and OP630378, respectively) and P02MS2A was identified as *Nigrospora oryzae* (accession number OP630041).

### Optimization of secondary metabolite production

Metabolite production was first optimized over 21 days following a method described by Thanabalasingam et al. [35]. Briefly, five fungal plugs (6 mm^2^) from a culture of each fungal isolate were inoculated into 200 mL of malt extract broth in a one litre Erlenmeyer flask (Pyrex®). Fungal cultures were sampled (5 mL) from day four of fermentation and with sampling every after two days until day 21. The samples were then extracted with an equal volume of methanol (ACE chemicals, South Africa), followed by overnight shaking at 150 rpm using a benchtop orbital shaker (Eins-Sci®). All samples were then filtered with sterile gauze and dried at 40 °C. The fungal whole broth extracts were re-suspended to a concentration of 400 μg/mL with 0.2% Dimethyl Sulfoxide (DMSO, Sigma Aldrich, South Africa). Control cultures include fungi-free broth which was extracted and treated in the same way as the experimental cultures to ensure that the observed activity was a result of fungal metabolites. Preliminary antimicrobial activity screening assays were performed following well-diffusion assays with test bacterium, *Staphylococcus aureus* ATCC 25923 and *Staphylococcus aureus* CS (CS: clinical strain) to determine optimum activity using the method described by Magaldi et al. [36]. The fungal whole broth extracts with optimum activity were observed on day 14 (Additional file 1, Figure S1; Additional file 2, Table S1) which was selected as the optimal growth period for maximum production of secondary metabolites. Experimental fungal cultures were then cultivated in duplicates and extracted following the same method and conditions as described for optimization. This was done to increase residue production so that enough residue was collected after the whole broth extraction procedure using methanol.

### Antimicrobial activity testing using well-diffusion assays

All bacteria used as indicator organisms in this study were donated by the National Health Laboratory Services (NHLS) at Inkosi Albert Luthuli Central Hospital, Durban, South Africa. These test bacterial isolates were vancomycin-resistant *Enterococcus* (VRE) 05114870, *Staphylococcus aureus* (*S. aureus*) ATCC 25923, *S. aureus* CS, methicillin resistant *S. aureus* (MRSA) 25775, MDR *Pseudomonas* 5625574, MDR *Acinetobacter baumannii* 5630947, and Carbapenem-resistant *Acinetobacter baumannii* (CRAB) 5630942. These bacterial clinical isolates were selected as test microbes based on their resistance profiles, and they have previously shown to be resistant to a variety of antibiotics, including ampicillin, tetracycline, methicillin, meropenem, trimethoprim, vancomycin among many other antibiotics [37, 38].

The antibacterial activities of fungal whole broth extracts were assessed by well diffusion assays following a protocol described by Magaldi et al. [36]. Briefly, bacteria were cultured in Muller Hinton broth (MHB, Merck, Darmstadt,Germany) overnight at 37ºC before they were standardized against a 0.5 McFarland standard (1.5 × 10^8^ colony forming units (CFU/mL). The bacterial suspension’s turbidity was measured at OD_600_ nm using an iMark Microplate Absorbance Reader (Bio-Rad, South Africa). After that, a sterile swab was dipped into the standardized culture, and excess fluid was removed by pressing the swab against the walls of the tube. The swab was then used to spread the culture evenly over the surface of respective MH agar plates, and the plates were left to dry for one minute. Thereafter, a sterile cork borer was used to drill wells into each MH agar plate. Then 80 μL of fungal whole broth extracts were added to wells at a concentration of 100 μg/mL. The extracts were allowed to diffuse for 10 min before the plates were incubated overnight at 37ºC. The diameters of zones of clearance were measured the following day. The negative control included fungal-free ME broth extracted in the same way as experimental cultures and the diluent, 0.2% DMSO was also included as a sterility control. The assays were done in duplicates and the fungal whole broth extracts that exhibited antibacterial activity against the test bacteria were selected for further assays.

### Minimum Inhibitory Concentrations (MICs)

The microtiter broth dilution method was used to assess the minimum inhibitory concentrations (MICs) of the fungal whole broth extracts that exhibited bioactivity against indicator bacteria isolates [39] following the Clinical Laboratory Standard Institute (CLSI) standards [40]. Briefly, 50 µL of Muller Hinton (MH) broth was added horizontally in rows from columns two to 12 on a 96-well flat bottom plate, with column 12 serving as a sterility control. Thereafter, 100 μL of fungal whole extracts were added to the first column. This was followed by a two-fold dilution of the extract by pipetting 50 µL from column 1 and serially diluting down to column 11, then discarding the withdrawn solution from column 11. The positive control used was ciprofloxacin while the negative control was a methanol extract from fungi-free MEB broth (resuspended in 0.2% DMSO). Bacterial indicator organisms were cultured and standardized using a 0.5 McFarland standard as described in the well-diffusion assay. After that, the bacterial cells were diluted to achieve a bacterial suspensions of approximately 1 × ${10}^{6}$ CFU/mL. Then 50 µL of the respective bacterial suspension was added to all wells and the plates were covered and incubated at 37ºC for 24 h. The final concentration ranged from 256 μg/mL to 0.125 μg/mL.

After 24 h of incubation, the MICs were determined using iodonitrotetrazolium chloride (INT, Sigma Aldrich, South Africa) as an indicator of the presence or absence of live bacterial cells. The INT assay was done by adding 10 µL of 0.2 μg/mL INT to all wells of microtiter plates, then they were covered and incubated at 37ºC for 30 min. Bacterial growth was observed by a pink color change indicative of a reaction of live bacteria able to reduce INT to iodonitrotetrazolium formazan.

### Solid-Phase Extraction (SPE) method coupled with Gas Chromatography-Mass Spectrometer analysis (GC–MS)

The whole broth extracts of fungal endophytes that exhibited bioactivity were then submitted for partial purification by solid-phase extraction (SPE) as described by Cutignano et al. [41] with some modifications. Briefly, whole broth extracts were diluted in 50% methanol to a concentration of 100 μg/mL. The Carbon 18 (C18) cartridges (Sigma Aldrich, South Africa) were attached to the SPE vacuum manifold and conditioned with 3 mL of 100% HPLC grade methanol (Sigma Aldrich, South Africa). The C18 sorbent cartridges were then equilibrated with 3 mL of sterile distilled water. After equilibration, 3 mL of each whole broth extract was added into the respective C18 sorbent SPE cartridges, and all samples were slowly passed under vacuum for not more than 20 pgs.

This was followed by a series of elution steps which included the following; fraction A: 6 mL of sterile distilled water (${\mathrm{H}}_{2}\mathrm{O}$), fraction B: 9 mL of methanol–water (${\mathrm{CH}}_{3}$ OH/${\mathrm{H}}_{2}\mathrm{O})$ (50:50 *v/v*) (Sigma Aldrich®), fraction C: 9 mL of acetonitrile–water (${\mathrm{CH}}_{3}$ CN/${\mathrm{H}}_{2}\mathrm{O})$ (70:30 *v/v*) (Sigma Aldrich®), fraction D: 9 mL of 100% acetonitrile (${\mathrm{CH}}_{3}$ CN) (LiChrosolv®) and fraction E: 9 mL of dichloromethane-acetonitrile $({\mathrm{CH}}_{2}{\mathrm{CL}}_{2}$/${\mathrm{CH}}_{3}\mathrm{OH}$) (90:10 *v/v*) (Sigma Aldrich®). All fractions were collected into their respective elution tubes.

The eluted fractions were then concentrated by evaporation to dryness under a gentle stream of nitrogen. The dry fractions were reconstituted with 1 mL of 0.2% DMSO (for bioactivities) or HPLC-grade dichloromethane (for GC–MS analysis). The fractions were then vortex at maximum speed for 15 s, before bioactivity assays following the method described by Magaldi et al. [36] to assess the bioactivity of the fractions. Active fractions as observed in the well-diffusion assays were further analyzed using Gas Chromatography-Mass Spectrometer (GC–MS) at the Department of Chemistry, University of KwaZulu-Natal, Pietermaritzburg campus.

For GC–MS analysis, each sample was splitless injected (split 20:80–8-200-5 M-8–260-10M10-280-HP5) on a Hewlett Packard 6890 (USA) gas chromatography. The samples were separated using an Agilent 19091S—433 column (30 m x 250 m × 0.25 m). The starting column temperature was 35 °C with a three-minute hold time. The temperature was set to increase at 8 °C/min, with a maximum temperature of 280 °C. One microliter of the sample was injected into the port, vaporized, and transported down the column at a flow rate of 1 mL/min using helium as the carrier gas. The MS Spectrum was captured at 70 eV. The components were identified and evaluated using a Flame Ionization Detector (FID) after separation in the column (Mishra and Patnaik, 2020). Compounds were identified by comparing the spectrum of unknown compounds to the spectrum of known compounds in the National Institute of Standards and Technology (NIST MS 2.0) structural library to determine their names, molecular weight, and structure [42].

## Results and discussion

### Dereplication and identification of fungal endophytes

**Fig. 1:**
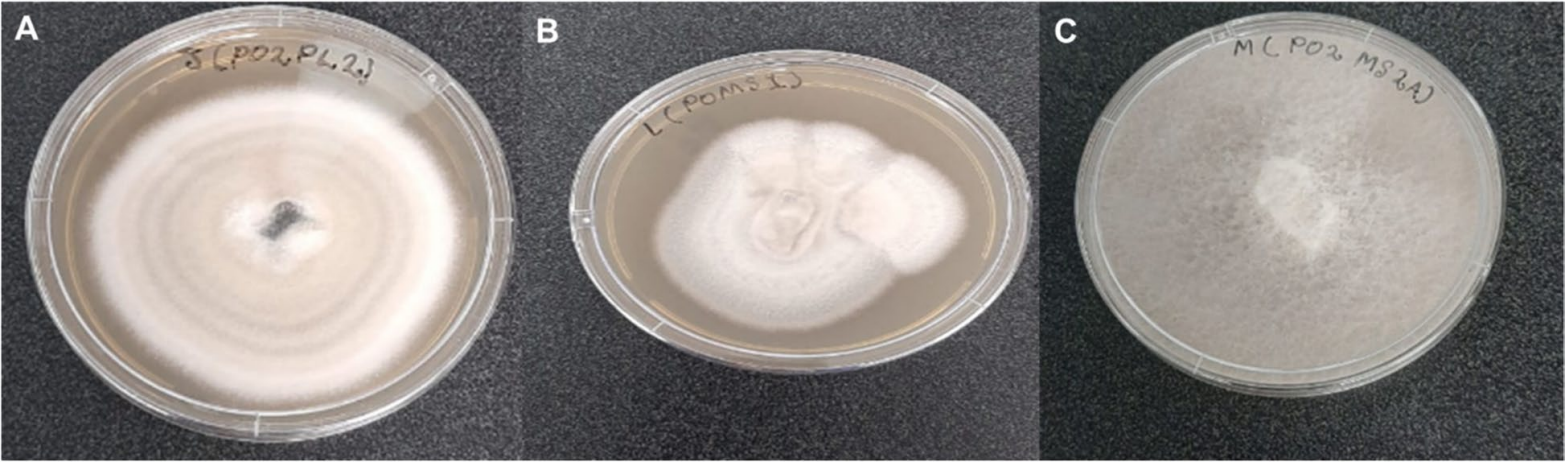
Macroscopic identification of bioactive endophytic fungi isolated from *Sclerocarya birrea*. **A**. shows mycelia growth of fungal endophyte isolate P02PL2, **B**. shows mycelia growth of fungal endophyte isolate P01MS1, and **C**. shows mycelia growth of fungal endophyte isolate P02MS2A

Preliminary bioactivities were conducted with whole broth extracts from the ten endophytic fungal isolates and only three fungal endophytes, P02PL2, P02MS1, and P02MS2A exhibiting bioactivity against a panel of indicator bacteria were considered for further evaluation (Fig. 1). Microscopic identification showed that all the endophytic fungal isolates had similar microscopic characteristics, as they all appear to have conidia. This means that these isolates are spore-forming fungi. All three bioactive fungal endophytic isolates were subjected to molecular identification based on internal transcribed spacer (ITS) region sequencing.

The amplification of a 534 bp fragment was achieved using an internal transcriber space polymerase chain reaction (ITS-PCR). The ITS region of ribosomal DNA is a successful de factor barcode for fungal endophytes [43]. In fungi, the entire ITS region averages between 500 and 600 base pairs (bp) for ascomycetes and basidiomycetes, respectively [44]. The BLAST search results for the consensus sequences resulted in varying fungal genera with identities of and sequence coverages of 96%—100% (Table 1).

**Table 1:** Morphological and molecular characterization of endophytic fungal isolates

**Strain ID**	**Morphological characteristics**	**NCBI Blast match**	**Sequence ID (%)**	**Query coverage**	**NCBI accession number**
P02PL2	Connected conidia chain with some large spores and long septate	*Alternaria alternata*	100	100	OP630378
P02MS1	Septate hyphae with curved and straight conidiophores. Separate branches of chains with conidia appear to be obclavate. Also viewed in the shape of long ellipsoids	*Alternaria alternata*	100	98–100	OP6299950
P02MS2A	Septate hyphae, conidia with a spherical shape, and possesses branched conidiophores	*Nigrospora oryzae*	100	96–100	OP630041

### Antimicrobial activities of fungal endophytes whole broth extracts

In this study, we first optimized the production of secondary metabolites by isolated endophytic fungi. Maximum activity as observed by the diameter of the zone of clearing in the well diffusion assay was reached on day 14. Subsequent production of secondary metabolites was then performed for 14 days and only three out of the ten isolated endophytic fungi showed antibacterial activity against *S. aureus* ATCC 25923 and *S. aureus* C. S. Miethke et al. [5] recently suggested that positive hits selected from screening panels should also be tested against recently isolated drug-resistant clinical pathogens to profile their activity on existing resistance mechanisms. Therefore, we extended the testing panel by obtaining recently isolated multidrug-resistant clinical isolates listed by WHO as priority pathogens from the National Health Laboratory Services (NHLS) at Inkosi Albert Central Hospital in Durban.

The bioactivity of fungal whole broth extracts was tested against five bacterial isolates: vancomycin-resistant *Enterococcus* (VRE) 05114870, multidrug-resistant *Staphylococcus aureus* (MRSA) 25775, MDR *Pseudomonas* 5625574, MDR *Acinetobacter baumannii* 5630947, and carbapenem-resistant *A. baumannii* (CRAB) 5630942. All three fungal whole broth extracts showed strong bioactivity against two of the tested bacterial strains; gram-negative MDR *Pseudomonas* 5625574 and gram-positive MRSA 25775 (Table 2).

**Table 2:** Antibacterial activities of whole broth extract of endophytic fungal isolates. Whole broth extracts of *A. alternata* P02PL2, *A. alternata* P02MS1, and *N. oryzae* P02MS2A were tested against *S*. *aureus* ATCC 25923 (A), *S. aureus* C.S (B), MRSA (methicillin-resistant *S. aureus*) 25775 (C), and MDR (multidrug-resistant) *Pseudomonas* (D). The control included a fungi-free MEB whole broth extract), and the diluent, 0.2% dimethyl sulfoxide (DMSO). The Zone of inhibition sizes represents the mean values of triplicates from each fungal whole broth extract

**Bacterial ID**	**Zone of inhibition diameter (mm)**				
	**Fungal extracts bioactivities**			**Experimental controls**	
	***A. alternata*** ** P02PL2 extracts**	***A. alternata*** ** P02MS1 extract**	***N*** **. ** ***oryzae*** ** P02MS2A extract**	**Fungi-free extracts**	**0.2% DMSO**
***S*** **.** *** aureus*** ** ATCC 25923**	14 ± 0.8	13.7 ± 0.9	10.7 ± 0.5	-	-
***S. aureus*** ** CS**	9.7 ± 0.5	10.3 ± 0.9	11	-	-
**Carbapenem-resistant ** ***A. baumanii*** ** (CRAB) 5630942**	-	-	-	-	-
**Multidrug-resistant (MDR) ** ***A. baumanii*** ** 5630947**	-	-	-	-	-
**Vancomycin-resistant ** ***Enterococcus*** ** (VRE) 05114870**	-	-	-	-	-
**MRSA 25775**	11 ± 0.5	11.3 ± 1.2	9.7 ± 0.5	-	-
**MDR ** ***Pseudomonas *** **5625574**	10.7 ± 0.5	9.7 ± 1.2	13.7 ± 1.7	-	-

*ATCC* Amerlfoxideican Type Culture Collection,*CS* Clinical Strain, *MRSA* Methicillin Resistant *Staphylococcus aureus*, *MDR* Multidrug resistant, *DMSO* Dimethyl sulfoxide, *(−)* No activity

### Determination of minimum inhibitory concentrations

The fungal whole broth extracts showing activities against the panel of indicator bacteria were further probed for their minimum inhibitory concentration (MIC) (Table 3 and Additional file 1, Figure S2). The most notable MIC value was observed for the of *N*. *oryzae* P02MS2A whole broth extract against MDR *Pseudomonas* 5625574 at a concentration of 1.0 μg/mL, and for methicillin-resistant *S. aureus* (MRSA) 5627679, MIC was observed to be 16 μg/mL. The accepted MIC by CLSI ranges from 0.25 to 1.0 μg/mL [45]. Therefore, the MIC value of 1.0 μg/mL obtained in this present study means MDR *Pseudomonas* 5625574 showed susceptibility to *N. oryzae* P02MS2A whole broth extract. These results suggest that the isolated endophytic fungi *N*. *oryzae* P02MS2A has a potential to produce active compounds with broad-spectrum of activities even against contemporary clinical isolates exhibiting multidrug resistance. Minimum inhibitory concentration value of 32 μg/mL was observed for whole broth extracts for endophytic fungi: *A*. *alternata* P02PL2, and *N*. *oryzae* P02MS2A against *S. aureus* ATCC 25923. Several previous studies have reported evidence that endophytic fungi *A. alternata* synthesize bioactive compounds with significant antibacterial activity [46]. For instance, previous studies done by Katoch et al. [46] reported significant bioactivity exhibited against *S*. *aureus* by fungal whole broth extract from *A. alternata* isolated from *Monarda citriodora L.* The endophytic fungi *N. oryzae* has also been reported to synthesize noteworthy antimicrobial compounds [47].

**Table 3:** Minimum inhibitory concentrations shown by fungal endophytes whole broth extracts, Ciprofloxacin as positive control, and fungi-free extract as negative control. All fungal endophytes whole broth extracts were tested in duplicates and mean MICs were calculated

**Fungal endophytes**	**Tested bacteria strains, mean MICs (μg/mL)**			
	***S. aureus*** ** ATCC 25923**	***S. aureus*** ** C. S**	**MRSA 5627679**	**MDR ** ***Pseudomonas*** **5625574**
*A. alternata* P02PL2	32	64	32	256
*A. alternata* P02MS1	64	64	128	64
*N. oryzae* P02MS2A	32	32	1	16
Ciprofloxacin	-	-	-	-
Fungi-free extract	-	-	-	-

*ATCC* Amerlfoxideican Type Culture Collection, *CS* Clinical Strain, *MRSA* Methicillin Resistant *Staphylococcus aureus*, *MDR* Multidrug resistant; (−): no activity, *MICs* Minimum inhibitory concentrations

Nevertheless, the *N*. *oryzae* P02MS2A whole broth extract also exhibited bioactivity at MIC of 32 μg/mL against *S. aureus* CS. Similar results were observed on crude extracts of *A. alternata* P02PL2 against MRSA 5627679. The endophytic fungi: *A. alternata* P02PL2 exhibited MIC values at concentration of 64 μg/mL against *S. aureus* CS, and *A*. *alternata* P02MS1 against *S. aureus* ATCC, *S. aureus* CS, and MDR *Pseudomonas* 5625574. The fungal endophyte *A*. *alternata* P02MS1 also exhibited an MIC of 128 μg/mL against MRSA 5627679. However, the highest MIC value was observed at 256 μg/mL for fungal endophyte *A*. *alternata* P02MS1 against MDR *Pseudomonas* 5625574. All these MIC results suggest that these fungal endophytes exhibited weaker antibacterial activities against the panel indicator bacteria used in this study. Arivudainambiet et al. [48] have also reported high MIC values for crude extracts of endophytic fungi *A*. *alternata* with the lowest MIC value at 200 μg/mL against *S. aureus*. While it is emphasized that MIC of 32 μg/mL is a breaking point of resistance, and any MIC equal or greater than MIC of 32 μg/mL means that the tested bacterial strain is resistant to potential antimicrobial compound that is tested. Therefore, in this study, the MIC values of 32 μg/mL, 64 μg/mL, 128 μg/mL, and 256 μg/mL were interpreted as resistance by tested bacteria isolates over potential antibacterial compounds.

However, it should be considered that cultivation of environmental microorganisms such as the fungal endophytes reported here, under laboratory conditions often leads to silencing or production of low concentrations of bioactive compounds [49]. As a consequence, several genomic and cultivation based strategies has been proposed to stimulate the expression of these biosynthetic pathways even in axenic culture conditions [50]. In addition, the complex chemical space in fungal crude extracts may mask the action of the bioactive compound resulting in low activities. Thus, all the potential activities observed here could potentially be enriched with isolation and purification steps [51, 52].

### Solid-Phase Extraction (SPE) and Gas Chromatography-Mass Spectrometer (GC–MS) analysis

#### Solid-phase extraction and well diffusion assay

The crude extracts obtained from fungal secondary metabolites production results in complex mixtures that make it difficult to identify the compound responsible for bioactivity [52]. Dereplication using chromatographic analysis hyphenated to mass spectrometry followed by similarity searches on databases is often necessary to decipher the chemical space in the complex chemical mixtures [52]. In this study, we used solid phase extraction (SPE) to concentrate and partially purify the complex fungal crude extracts into enriched fractions with reduced chemical space complexity. The SPE protocol described by Cutignano et al. [41] was used which employs a series of solvent mixtures with increasing chromatographic strengths. First, the fungal crudes extracts were washed with distilled water to remove amino acids and saccharides. Following this, the extract was treated with a mixture of water and methanol (50:50; v/v) to elute nucleosides followed by partitioning in a mixture of acetonitrile and water (70:30; v/v) to elute glycolipids and phospholipids, acetonitrile to elute free fatty acids and dichloromethane acetonitrile mixture (90:10; v/v) to elute triglycerides.

After the partial purification of extracts by well diffusion assay, the fractions were assessed for bioactivity using well diffusion assays (Table 4). The results revealed that Fraction B of *A. alternata* P02PL2 exhibited bioactivity against *S*. *aureus* ATCC 25923, and clinical isolates, *S. aureus* CS, MRSA 25775, and MDR *Pseudomonas* 5625574. Fraction E of *A. alternata* P02PL2 exhibited bioactivity against *S*. *aureus* ATCC 25923. A similar activity pattern to Fraction B of *A. alternata* PO2PL2 was observed with Fraction B and C of *A*. *alternata* P02MS1 and Fraction B of *N. oryzae* P02MS2A. No bioactivities were observed for the fungi-free extract which was used as a control in this study. All bioactive fractions were submitted for characterization by GC–MS analysis.

**Table 4:** Antimicrobial activities exhibited by Fractions from bioactive fungal endophytes whole broth against at least one of the tested microorganisms; *S. aureus* ATCC 25923, *S. aureus* CS, MRSA 25775, and MDR *Pseudomonas* 2625374

**Bacterial ID**	**Fungal endophytes crude extract fractions** **Zone of inhibition diameter (mm)**																	
	***A. Alternaria*** ** P02PL2**						***A. alternaria*** ** P02MS1**						***N. oryzae*** ** P02MS2A**					
	**FA**	**FB**	**FC**	**FD**	**FE**	**Fungi-free extract**	**FA**	**FB**	**FC**	**FD**	**FE**	**Fungi-free extract**	**FA**	**FB**	**FC**	**FD**	**FE**	**Fungi-free extract**
***S*** **.** *** aureus*** ** ATCC 25923**	-	8	-	-	11	-	-	12	13	-	-	-	-	9	-	-	-	-
***S. aureus*** ** CS**	-	9	-	-	-	-	-	9	9	-	-	-	-	8	-	-	-	-
**MRSA 25775**	-	8	-	-	-	-	-	9	8	-	-	-	-	9	-	-	-	-
**MDR ** ***Pseudomonas *** **5625574**	-	8	9	-	-	-	-	8	9	-	-	-	-	9	-	-	-	-

*FA* *−* *E* Fractions A to E, *(−)* No activity, *ATCC* American Type Culture Collection, *CS* Clinical Strain, *MRSA* Methicillin Resistant *Staphylococcus aureus*, *MDR* Multidrug resistant

#### Gas Chromatography-Mass Spectrometer (GC–MS) analysis

**Fig. 2:**
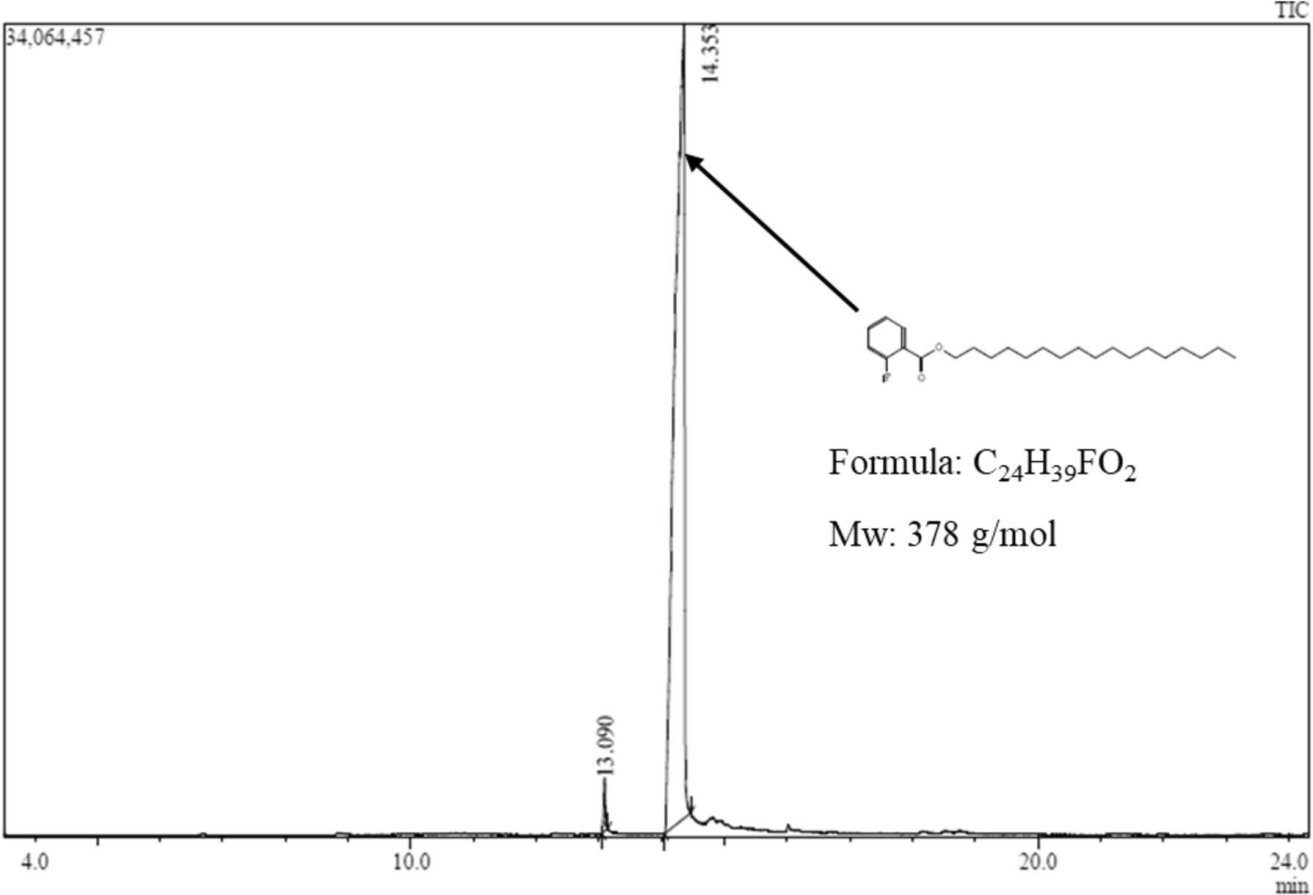
GC–MS chromatogram illustration of compounds detected contained on Fraction C of *A*. *alternata* P02MS1 whole broth extract. The peak points (elution time 13.09 and 14.353) were putatively identified as one compound (2-fluorobenzoic acid heptadecyl ester). The compound molecular weight (Mw): 378 g/mol

Gas-Chromatography-Mass Spectrometer was selected for chemical profiling of the fractions exhibiting activities in antimicrobial assays. Although liquid chromatography hyphenated to mass spectrometry (LC–MS) is a dominant analysis method for separating microbial crude extract mixtures to cover a large range of secondary metabolites, GC–MS can also be an efficient choice to cover chemical space of small compounds that can be derivatized to heat stable analytes [51]. In addition, GC–MS provides superior separation ability and reproducible EI^+^ ionization and dynamic range, and universal mass spectral library for small molecular weight compounds [51–53]. A visual inspection of the resulting mass chromatogram profiles characterized a total of 78 compounds from five fractions of three endophytic fungi. In the fractions derived from *A. alternata* P02PL2, 12 compounds were detected for Fraction B (Additional file 1, Figure S2 and Additional file 2, Table S2, and 35 compounds for Fraction E (Additional file 1, Figure S3 and Additional file 2, Table S3. For *A*. *alternata* P02MS1, 22 compounds were detected in Fraction B (Additional file 1, Figure S4 and Additional file 2, Table S4, and 2 compounds in Fraction C (Fig. 2 and Additional file 2, Table S5). Seven compounds we detected in Fraction B of *N*. *oryzae* P02MS2A (Additional file 1, Figure S5 and Additional file 2, Table 6).

Nine of the compounds in these chemical profiles have been reported in previous studies to exhibit antimicrobial or antioxidant properties. Three unique bioactive compounds of the chemical compound; Pyrrolo[1,2-a]pyrazine-1,4-dione, hexahydro-3- (Additional file 2, Table S2) were detected from Fraction B of *A. alternata* P02PL2 have been previously shown to exhibit antioxidant activity, associated with the reduction of oxidative stress associated with the neurodegenerative diseases such as Parkinson’s diseases and Alzheimer’s disease [54, 55]. Another bioactive compound is Benzene, 1,3-bis (1,1-dimethylethyl) observed in Fraction B fractionated from *A. alternata* P02PL2, was previously extracted by hydro distillation from *Parkinsonia aculeate* plant’s leaves and shown to exhibit notable antibacterial and antifungal activities [56]. The Fraction B and E of *A. alternata* P02PL2 were detected to have a common compound, n-Hexadecanoic acid at retention time of 18.6 min in both fractions. The n-Hexadecanoic acid is a fatty acid (palmitic acid) [57], that has antioxidant, hypocholesterolaemia, and pesticides properties [58], anti-inflammatory effect [59], antibacterial [60], antifungal [61], and anticancer effects [62].

In Fraction C of *A*. *alternata* P02MS1 (Fig. 2), one putative bioactive compound (Fluorobenzoic acid heptadecyl ester) was detected as a pure compound split into two peaks. Since this fraction was active against MDR *Pseudomonas* 2625374 and MRSA 25775, we can deduce that the observed activities for this fraction are from this compound. Since this compound was also detected in other fractions active against MDR *Pseudomonas* 2625374 and MRSA 25775; Fraction E from *A. alternata* P02PL2, Fraction B, and C from *A*. *alternata* P02MS1, and Fraction E from *N*. *oryzae* P02MS2A, we infer that the observed activities in these fractions were likely due to the presence of this compound in these Fractions. The compound, 2-Fluorobenzoic acid. heptadecyl ester has been previously isolated from endophytic fungi *A. alternata* isolated from *Lawsonia inermis Linn* [63]. However, no individual compound in this fungal whole broth extract was associated with these activities. Instead, this study identified 2-fluorobenzoic acid heptadecyl ester in the GC–MS profile of various fractions and showed this compound to be associated with antibacterial properties [63]. The noteworthy biological activities of other compounds detected from the *A. alternata* P02PL2, *A*. *alternata* P02MS1 and *N*. *oryzae* P02MS2A fractions are summarized in Table 5. Although we could elucidate a wide chemical space using the GC–MS technique, the authors acknowledge that the addition of LC–MS spectral data would allow for a inclusive impression and comprehensive coverage of the chemical space in these fractions.

**Table 5:** Chemical profiles of selected compounds with assigned bioactivities obtained from the GC–MS fraction’s fingerprint

**Fungal endophyte**	**Fraction (F)**	**Compound Name**	**Retention Time (min)**	**Chemical Formula**	**Molecular** **Weight (g/mol)**	**Area %**	**Reported bioactivity**	**Nature of Compound**
*A. alternata* P02PL2	FB	Benzene, 1,3-bis(1,1-dimethylethyl)	9.930	C_14_H_22_	190	2.06	Antibacterial activity [56]	Aromatic hydrocarb
		Pyrrolo[1,2-a] pyrazine-1,4-dione, hexahydro-3-(2-methylpropyl)-	16.547, 18.035, and 18.685	C_11_H_18_N_2_O_2_	210	6.85, 1.53, & 0.85	antioxidant activity [54], antibacterial activity [64]	alpha-amino acid
*A. alternata* P02PL2	FE	Butylated hydroxyanisole	12.792	C_15_H_24_O	220	0.92	Antioxidant [65]	Phenol
		2-Fluorobenzoic acid. Heptadecyl ester	14.111	C_24_H_39_FO_2_	378	10.09	Antiangiogenic properties [63]	Benzoic acid
		Eicosane	15.134	C_20_H_42_	282	0.70	Antibacterial activity [66]	Long chain fatty acid
		Dodecane, 2,6,11-trimethyl-	15.896	C_15_H_32_	212.41	0.60	Antibacterial activity [67]	Alkane
		n-Hexadecanoic acid	18.629	C_16_H_32_O_2_	256	3.59	See above	fatty acid
*A. alternata* P02MS1	FB	2-Fluorobenzoic acid. Heptadecyl ester	14.222	C_24_H_39_FO_2_	378	17.40	Antiangiogenic properties [63]	Benzoic acid
		17-Pentatriacontene	14.539	C_35_H_7_O	490	5.73	Antioxidant activity [68]	Alkene
*A. alternata* P02MS1	FC	2-Fluorobenzoic acid. Heptadecyl ester	13.090	C_24_H_39_FO_2_	378	1.12	Antiangiogenic properties [63]	Benzoic acid
*N. oryzae* P02MS2A	FB	1-Heptanol, 2,4-dimethyl	10.612	C_11_H_24_O	172	1.48	Antifungal activity [69]	Long chain alcohol
		2-Fluorobenzoic acid. Heptadecyl ester	14.213	C_24_H_39_FO_2_	378	21.59	Antiangiogenic properties [63]	Benzoic acid

*GC* *–* *M* *S* Gas chromatography−mass spectrometry, *FA, B, C and E* Fraction A, B, C and E)

In this study, all Fractions from *A*. *alternata* and *N*. *oryzae* exhibiting antimicrobial activity against four-panel indicator organisms: *S. aureus* ATCC 25923*, S. aureus C.S,* MRSA 25775, and MDR *Pseudomonas* 5625574 shared the compound, 2-fluorobenzoic acid heptadecyl ester*.* Therefore, our study shows that a putative 2-fluorobenzoic acid heptadecyl ester is an interesting compound with broad-spectrum antimicrobial properties against multidrug-resistant gram-positive (MRSA) and gram-negative (MDR *Pseudomonas*) pathogens listed in the WHO priority list of pathogens of interest with the potential to escape existing currently available antimicrobial regimen. It would be interesting to understand the mechanism of action of this compound, its toxicity in human cell lines, and to link the biosynthetic pathway responsible for its synthesis in the fungal genome.

## Conclusion

In this study, three endophytic fungal isolates showed a diverse chemical profile with diverse compounds some of which have no previously reported biological activities and might represent some novel activities and mechanisms that were not captured by the employed bioactivity assays in this study. The lower MICs exhibited by whole broth extract of* N*. *oryzae* P02MS2A against gram-negative multidrug-resistant (MDR) *pseudomonas* 5625574 and gram-positive MRSA 5627679 indicate that the active compound in the crude extract is highly effective with broad-spectrum antimicrobial properties. The activity observed against contemporary gram-negative multidrug-resistant clinical isolate is encouraging since there has been slow progress for production of antibiotics active against gram-negative pathogens has been launched in the past decades [70, 71]. Since we putatively identified the compound 2-fluorobenzoic acid heptadecyl ester as a common compound among bioactive fractions, it would be interesting to further evaluate a pure form of this compound for cytotoxicity, pharmacokinetics, and in vivo bioactivity in future studies.

All procedures were conducted in accordance to the guidelines and the study was approved by the UKZN Biomedical research ethics committee, reference number: BREC/00002127/2020. The ethics committee of the University of KwaZulu Natal permitted the use of the plant specimen for research purposes.

### Supplementary Information

**Additional file 1:** **Figure S1. **Optimization of antibacterial activity production from whole broth crude extract of endophytic fungi isolated from *S. birrea*. (A) antimicrobial activity against *S. aureus*(ATCC25923) and (B) antimicrobial activity against *S. aureus*(C.S) for 20 days. The controls included control 1 (fungi-free MEB crude extract) and control 2 (0.2% DMSO).** Figure S2.** GC-MS chromatogram illustration of compounds detected contained on Fraction B of *A. alternata *P02PL2 whole broth extract.** Figure S3.** GC-MS chromatogram illustration of compounds detected contained on Fraction E of *A. alternata *P02PL2 whole broth extract.** Figure S4.** GC-MS chromatogram illustration of compounds detected contained on Fraction B of *A*.* alternata* P02MS1 whole broth extract.** Figure S5.** GC-MS chromatogram illustration of compounds detected contained on Fraction B of *N*.* oryzae* P02MS2A whole broth extract.

**Additional file 2: Table S1.** Antibacterial activity of whole broth crude extracts from selected endophytic fungi. **Table S2.** Putative compounds detected by GC-MS on bioactive Fraction B of A. alternata P02PL2 whole broth extract. **Table S3.** Putative compounds detected by GC-MS on bioactive Fraction E of A. alternata P02PL2 whole broth extract. **Table S4.** Putative compounds detected by GC-MS on bioactive Fraction B of A. alternata P02MS1 whole broth extract. **Table S5.** Putative compounds detected by GC-MS on bioactive Fraction C of A. alternata P02MS1 whole broth extract. **Table S6.** Putative compounds detected by GC-MS on bioactive Fraction B of N. oryzae P02MS2A whole broth extract.

## Data Availability

All dataset generated and analysed during the current study are included in the Additional files 1 and 2. The voucher specimen of *Sclerocarya birrea* is publicly available at the Life Science Herbarium, University of KwaZulu Natal.
