# Supplementary material for: Endophytic fungal species Nigrospora oryzae and Alternaria alternata exhibit antimicrobial activity against gram-positive and gram-negative multi-drug resistant clinical bacterial isolates
Source: BMC Complement Med Ther. 2023 Sep 15;23:323. doi: 10.1186/s12906-023-04157-8 (PMC10504728; doi:10.1186/s12906-023-04157-8)
Supplement: Supplementary file 1 — Additional file 1: Figure S1. Optimization of antibacterial activity production from whole broth crude extract of endophytic fungi isolated from S. birrea. (A) antimicrobial activity against S. aureus(ATCC25923) and (B) antimicrobial activity against S. aureus(C.S) for 20 days. The controls included control 1 (fungi-free MEB crude extract) and control 2 (0.2% DMSO). Figure S2. GC-MS chromatogram illustration of compounds detected contained on Fraction B of A. alternata P02PL2 whole broth extract. Figure S3. GC-MS chromatogram illustration of compounds detected contained on Fraction E of A. alternata P02PL2 whole broth extract. Figure S4. GC-MS chromatogram illustration of compounds detected contained on Fraction B of A. alternata P02MS1 whole broth extract. Figure S5. GC-MS chromatogram illustration of compounds detected contained on Fraction B of N. oryzae P02MS2A whole broth extract. [file 12906_2023_4157_MOESM1_ESM.docx]

**Title: Endophytic fungal species *Nigrospora oryzae* and *Alternaria alternata* exhibit antimicrobial activity against gram-positive and gram-negative multi-drug resistant clinical bacterial isolates**

Authors: Asiphe Fanele and Sizwe I. Ndlovu


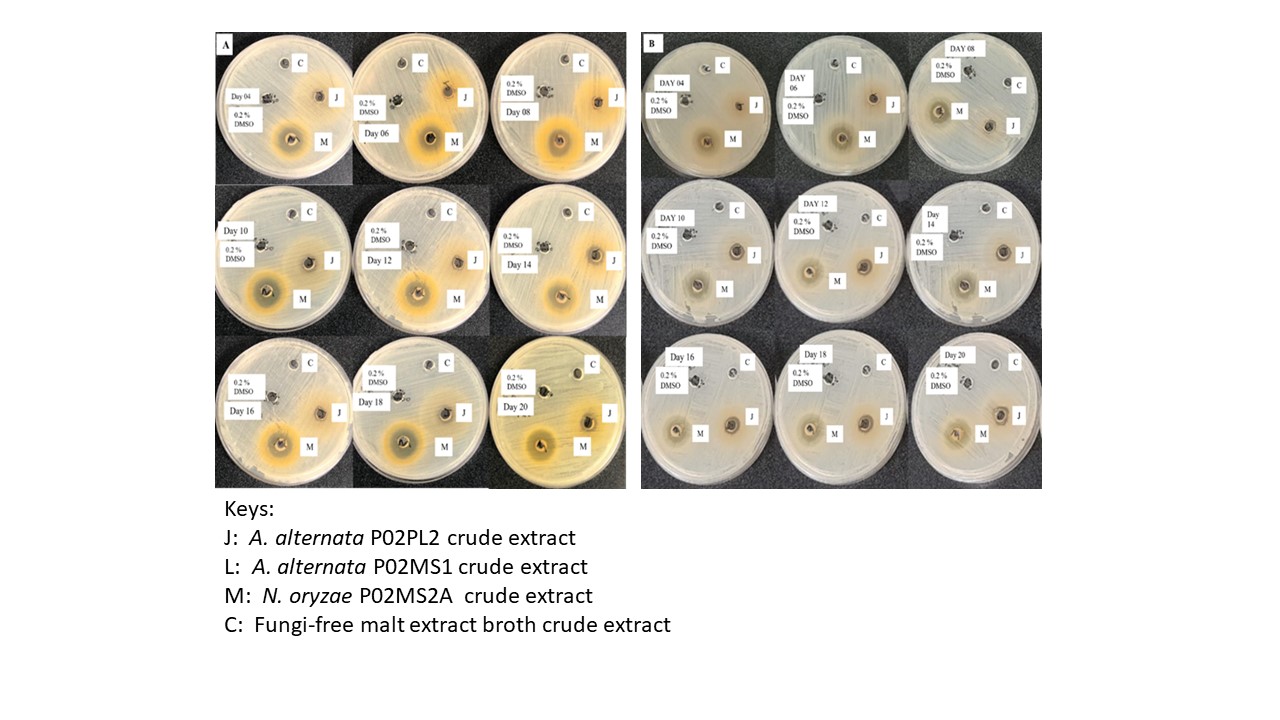


**Figure S1:** Optimization of antibacterial activity production from whole broth crude extract of endophytic fungi isolated from *S. birrea*. (A) antimicrobial activity against *S. aureus* (ATCC25923) and (B) antimicrobial activity against *S. aureus* (C.S) for 20 days. The controls included control 1 (fungi-free MEB crude extract) and control 2 (0.2% DMSO).


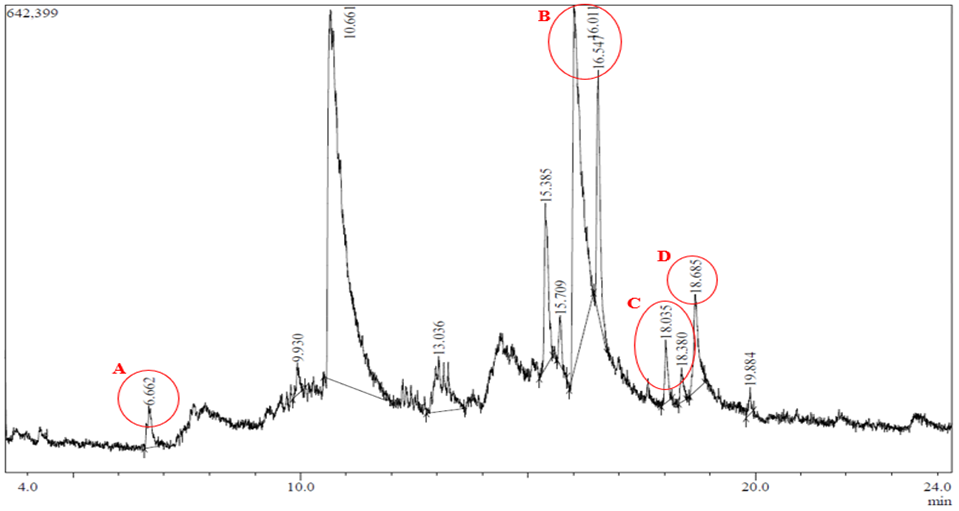


**Figure S2**: GC-MS chromatogram illustration of compounds detected contained on Fraction B of *A. alternata* P02PL2 whole broth extract.


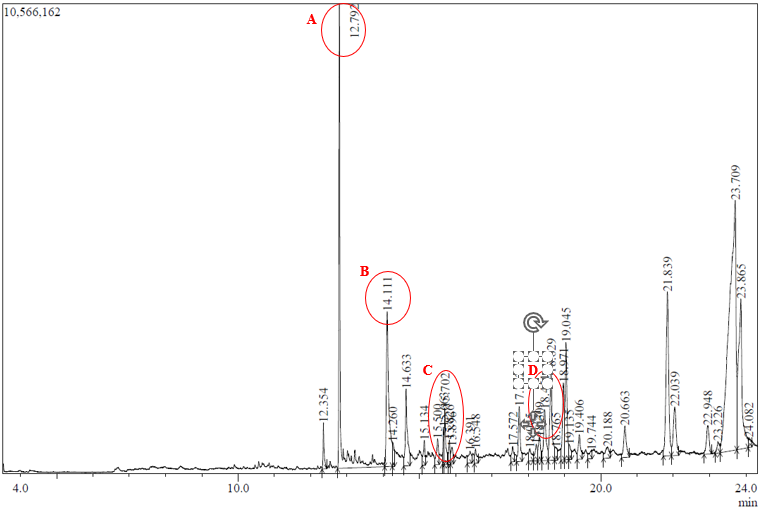


**Figure S3**: GC-MS chromatogram illustration of compounds detected contained on Fraction E of *A. alternata* P02PL2 whole broth extract.


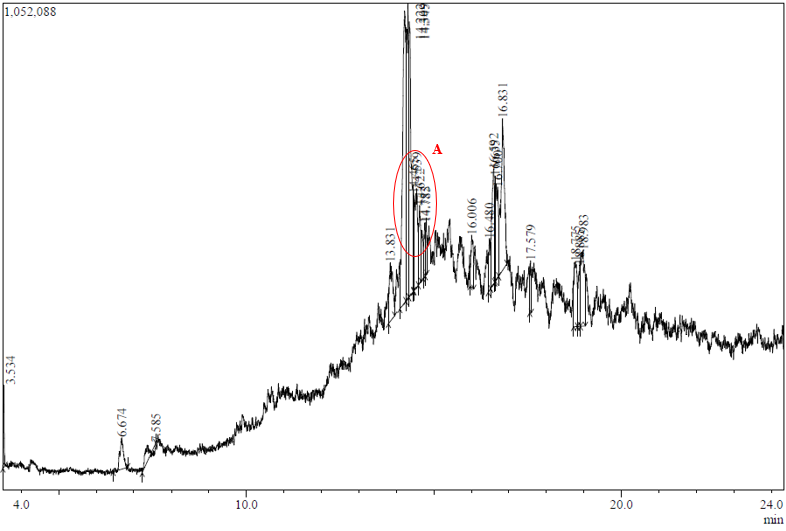


**Figure S4:** GC-MS chromatogram illustration of compounds detected contained on Fraction B of *A*. *alternata* P02MS1 whole broth extract.


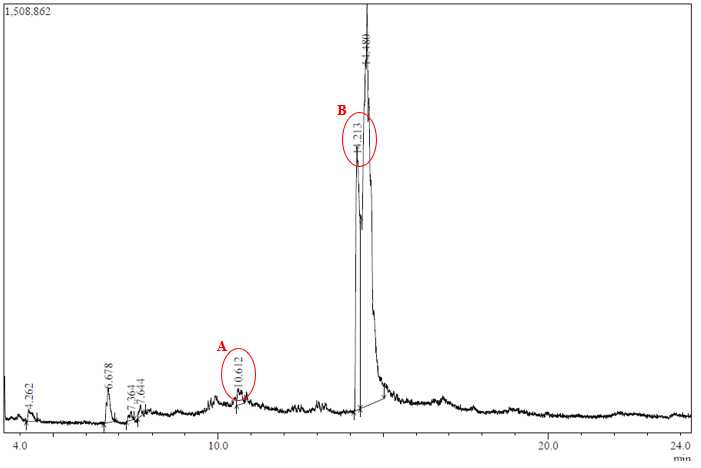


**Figure S5**: GC-MS chromatogram illustration of compounds detected contained on Fraction B of *N*. *oryzae* P02MS2A whole broth extract.
